# Annotations

**Published:** 1905-03-18

**Authors:** 


### ANNOTATIONS.

### The Government and Revaccination.

THE question of the revaccination of children at school age was raised in the House of Lords on Friday night by the Duke of Northumberland, but his attempt to induce the Government to make any move in the matter was quite futile. Lord Kenyon, in intimating that Ministers do not intend to introduce a measure, fell back for justification upon the report of the Royal Commission of 1896, which stated that compulsory revaccination was not practicable. He declared that as long as the conscientious objector is allowed to exist, it is impossible to bring in a Bill dealing fully and completely with vaccination and revaccination, and confessed that he was only in a position to express the hope that "the gradual diminution of cases which has been going on since the Act of 1898 came into operation, will continue until small-pox has almost died out of the land." Nothing could be more lame and impotent than this. Why is it that difficulties, which can be overcome in Germany, are insurmountable here? The Duke of Northumberland showed, in a sentence, the effect of hanging up the question of revaccination. From February 1904 to July 1905 there were 7,704 cases of small-pox in 315 urban districts, and of these upwards of 7,000 occurred in a comparatively small number of districts where vaccination and revaccination are not properly carried out. The truth is that the Government are afraid of the friends of the conscientious objector. On the same night that they declined to accede to the Duke of Northumberland's suggestion, the House of Commons passed, by a large majority, the second reading of a Bill to legalise Trade Union intimidation, it being pretty clear from the speeches of some members and the silence of others that, in view of the general election, it is considered necessary to curry favour with these organisations. Ministers, also influenced by the shadow of Dissolution, have arrived at the conclusion that of two evils it is better to incur the responsibility of chancing the recrudescence of a loathsome malady than to run the risk of losing a few votes at the polling booths. When compulsory vaccination was done away with, the Government virtually pledged themselves to introduce a Bill to encourage revaccination, but now they can only indulge in the aspiration that small-pox will almost die out, in spite of the conscientious objector being permitted to ignore the single safeguard against infection.

### The Purity of Drugs.

IN a recent address to the Pharmaceutical Society, Mr. Glyn-Jones discussed the public measures needed to secure the purity of drugs, and, amongst other suggestions, proposed the creation of a department of Public Health to be charged with the administration of the laws relating to adulteration. Arguing that in many cases the retailer is entirely innocent, not only of the act of adulterating, but also of the fact that the article sold was not pure, he urged that the object of legislation and administration should be rather to attack the source of the adulteration than, as at present, to make the retailer a scapegoat. Thus he would allow the retailer to establish a successful defence on proving that he had taken all reasonable precautions against committing an offence, that on demand made on behalf of the prosecutor he had given all information in his power with respect to the sources of supply, and that otherwise he had acted innocently in the matter. In considering these suggestions it must be remembered that the object of the law in the matter of adulteration is the protection of the public rather than the punishment of individuals, and it may be doubted whether this end would not be imperilled by the proposals suggested. Again, so far as drugs are concerned, the pharmacist professes to hold a diploma of competence, and it rests with him to determine the quality of the materials in which he deals by the application of the tests provided in the Pharmacopœia. Indeed it may be said that this is one of the main functions of that volume. It not only names the official drugs and defines their several sources, but it provides standards by which the genuineness of any sample may be tested. The pharmacist has therefore a full opportunity to secure the purity of his drugs, and if he fails to use this opportunity it is in the public interest that he should suffer for his neglect.

### The Multiplication of Discussions.

AMONG the various advantages which may be expected to follow the amalgamation of the medical societies at present existing in the metropolitan area is the diminution of the habit of repeated and largely useless discussions. There seems to be a growing tendency on the part of those who organise medical societies to believe that it is their duty at frequent intervals to throw into the arena some subject for debate and to enlist in the struggle a number of more or less well-known names. It may be allowed that there are questions which admit of this treatment. But they are not numerous, and perhaps still less numerous are the speakers fitted to deal amply with them. In many instances what happens is that after a more or less anxious gestation the officials of the society bring forth a subject for discussion and nominate a list of speakers. These may or may not include the one or two members of the profession fitted to estimate and to speak with authority on any modern development concerning the subject to be debated. But almost invariably there will appear in the official list several who can do nothing but present a re-hash of what is already perfectly well known, and who feel in the competitive spirit of the day a reason, or at least an excuse, for making as frequent an appearance as possible. The so-called debate is thus largely reduced to a chronicle of small beer, the staleness of which keeps the initiated in the shelter of their own homes. The reality of the performance is still further minimised by the fact that, so far from being a real debate or discussion, it consists of a series of prepared papers, each one of which is read without any necessary relationship to the other members of the series. The "puff preliminary" which appears in the Press and the prolonged report of the proceedings suggest an importance quite out of proportion to the real value of the scheme, but individual ambitions are flattered and the society seems to have attained a triumph. The relationship of all this to the extension or diffusion of knowledge is, however quite another story.

